# Decisions on Non-oncology Breakthrough Therapy Designation Requests in 2017–2019

**DOI:** 10.1007/s43441-023-00589-z

**Published:** 2023-11-05

**Authors:** Atasi Poddar, Miranda Raggio, John Concato

**Affiliations:** 1 Office of Medical Policy, Center for Drug Evaluation and Research, US Food and Drug Administration, WO51-6324, 10903 New Hampshire Avenue, Silver Spring, MD 20993 USA; 2 Office of New Drugs, Center for Drug Evaluation and Research, US Food and Drug Administration, Silver Spring, MD USA

**Keywords:** United States Food and Drug Administration, Drug development, New drugs, Investigational drugs, Biological products

## Abstract

**Background:**

The US Food and Drug Administration’s Breakthrough Therapy Designation (BTD) program is intended to facilitate and expedite development of investigational drugs to address unmet medical needs. The objective of this study is to provide an update on FDA’s process for review of BTD requests.

**Methods:**

We reviewed Center for Drug Evaluation and Research (CDER) decisions to grant or deny breakthrough therapy designation requests for non-oncology drugs or biological products (“drugs”) from January 1, 2017, through December 31, 2019. Data collection included characteristics of the corresponding drug and condition, reasons for granting or denying breakthrough therapy status, reasons for rescinding or withdrawing breakthrough therapy status after a request was granted (if applicable), and subsequent marketing approval status through 2022.

**Results:**

Among 240 requests, 93 (39%) requests were granted and 147 (61%) requests were denied. Granting of requests was more common for conditions where no therapy was available or for orphan diseases. Common reasons for denial included data-related issues, insufficient treatment effect, inadequate study design, endpoint attributes, safety issues, and reliance on post hoc analyses. Among 28 drugs receiving marketing approval as of the end of 2022 for the indication for which BTD was previously granted, 21 (75%) involved a first-in-class mechanism of action.

**Conclusions:**

This analysis describes CDER’s decision-making process related to review of requests for breakthrough therapy designations and enhances public awareness regarding efforts to expedite drug development.

**Supplementary Information:**

The online version contains supplementary material available at 10.1007/s43441-023-00589-z.

## Introduction

Breakthrough therapy designation (BTD) is an expedited program used by the Center for Drug Evaluation and Research (CDER) at the Food and Drug Administration (FDA) to accelerate the development and review of promising investigational drugs (i.e., human drugs and biological products) for serious and life-threatening diseases. Introduced in 2012 under Section 902 of the Food and Drug Administration Safety and Innovation Act (FDASIA) [21 U.S.C. 356(a)]—and further described (with pertinent definitions) in corresponding FDA guidance [1]—the criteria for BTD state that FDA will expedite the development and review of a drug “[…] if the drug is intended, alone or in combination with 1 or more other drugs, to treat a serious or life-threatening disease or condition and preliminary clinical evidence indicates that the drug may demonstrate substantial improvement over existing therapies on 1 or more clinically significant endpoints, such as substantial treatment effects observed early in clinical development” [2].

Sponsors can submit BTD requests concurrently with, or at any time after, an investigational new drug application (IND), and FDA then has 60 days to review and grant or deny the BTD request. The benefits of a BTD include more intensive FDA guidance on an efficient drug development program, an organizational commitment to involve senior managers in drug development and review, and eligibility for rolling review and priority review [1]. As a first step in the BTD review process, CDER Office of New Drug’s review divisions evaluate whether the BTD request fulfills three criteria: (1) data supporting the BTD request are from trials and corresponding investigational new drug applications (INDs) which are not on clinical hold; (2) the indication is for serious or life-threatening disease or condition; and (3) the clinical data provided by the sponsor are adequately and sufficiently complete to permit a substantive review. BTD requests that fails to fulfill any one of these criteria generally are denied at this initial step. For the remaining requests, a full review is then conducted by the relevant review division. The divisions’ planned determination is then reviewed by the Medical Policy and Program Review Council (MPPRC), a senior-level forum in CDER, to ensure that the policies to grant or deny BTD requests are applied consistently across CDER’s various therapeutic areas. In collaboration with the divisions, the MPPRC also discusses any new policy questions that may arise in the review of BTD requests to determine if any policy changes are appropriate.

In an earlier analysis [3], CDER described characteristics of the investigational drugs and related decisional factors used for granting or denying BTD among 315 requests received at CDER from July 2012 through June 2016. Prominent findings from the study include (1) during this earlier period, CDER granted 133 (37%), denied 182 (50%), and sponsors withdrew 49 (13%) requests before CDER made a decision, (2) the top three therapeutic categories for requests were for oncology/hematology, anti-viral, and neurology indications, (3) the majority of BTD requests included evidence from a single clinical phase 1 or 2 trial; (4) a lack of evidence supporting substantial improvement over existing therapies was the primary reason for denial and factors other than efficacy often contributed to this determination, (5) denials were often associated with multiple reasons.

This report represents an extension of the previous analysis, with a specific focus on non-oncology BTD decisions made during calendar years (CYs) 2017 through 2019, and with an end date of December 31, 2022, allowing for information to be gathered on the subsequent status of BTD marketing applications. Of note, the clinical settings and endpoints described in non-oncology BTD requests tend to be more diverse compared to oncology BTD requests, which tend to have more consistent endpoints (such as progression-free survival) applicable to various cancers. Accordingly, and by focusing on non-oncology indications, this analysis examines patterns in the evidence supporting granting of BTD across diverse conditions and endpoints, including factors that influenced a determination of sufficient preliminary clinical evidence that the drug may demonstrate substantial improvement over existing therapies on one or more clinically significant endpoints.

## Methods

### Description of the Data

For the present study, information about BTD requests was extracted from CDER’s Breakthrough Therapy Designation Determination Review Template (BTDDRT), BTD request decision letters, and other official documents archived in the *Document Archiving, Reporting, and Regulatory Tracking System* (DARRTS), which represents CDER’s official system of record. The data were collected by the primary reviewer (AP) and, when needed, secondary reviewers (JC and MR) contributed to the assessment of complex scenarios. Given that CDER does not disclose to the public the existence of an IND or any of its contents unless an IND has previously been disclosed publicly or acknowledged [4], only aggregate data from the BTD requests submitted under INDs and granted or denied between CY 2017 and CY 2019 are presented in this analysis.

**Figure 1:**
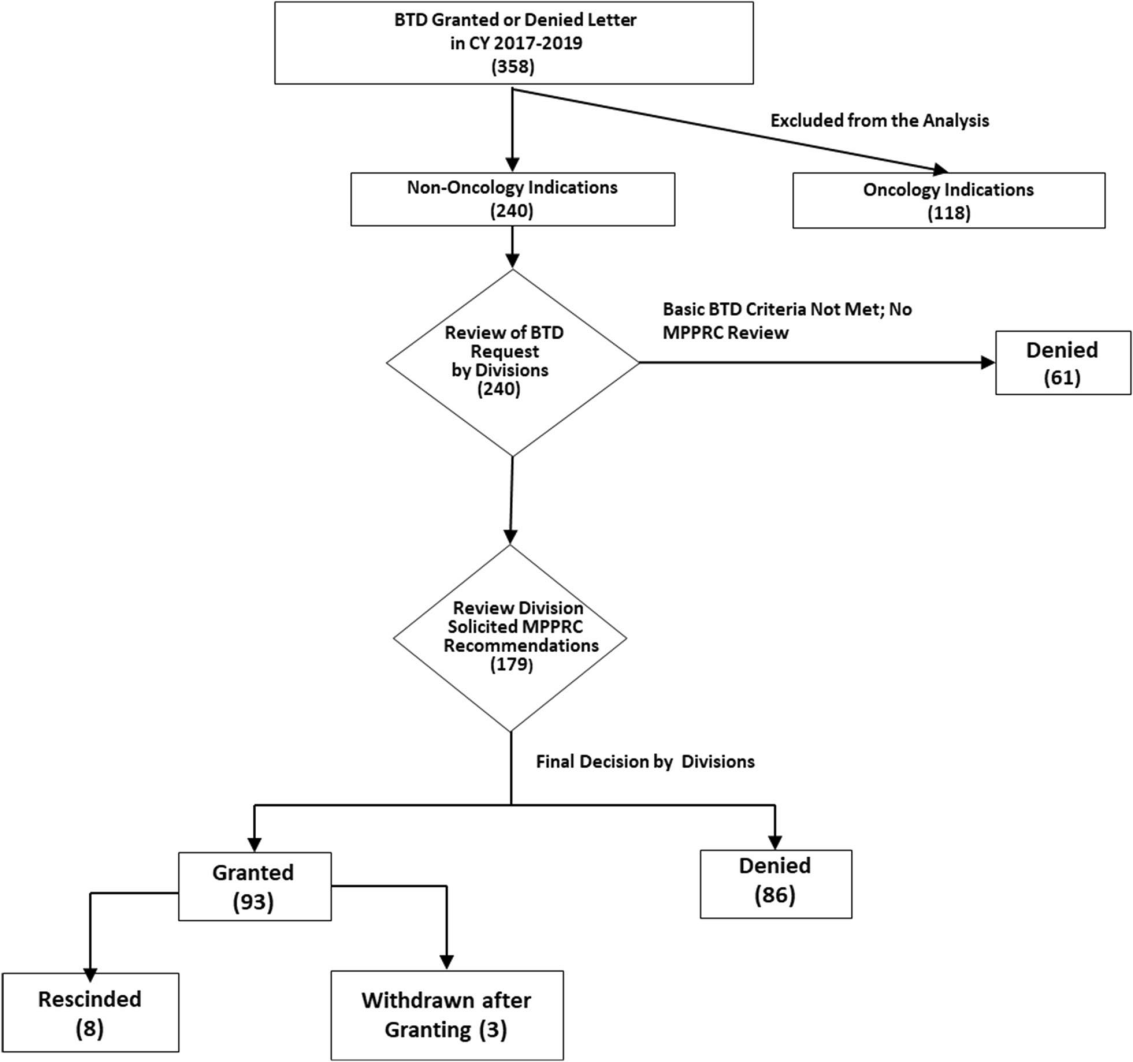
Actions Taken by CDER Review Divisions on BTD Requests.

Focusing on the decision date rather than receipt date for requests, CDER issued letters to grant or deny a total of 358 BTD requests from January 1, 2017, through December 31, 2019; see Fig. 1. Analyses for this report focused on the 240 (67%) requests involving non-oncology drugs. During the same time period, 51 additional BTD requests were withdrawn by the sponsor prior to CDER’s decision to grant or deny the request; these requests were not reviewed.

An orphan drug is intended for the treatment, prevention, or diagnosis of a rare disease or condition that affects less than 200,000 persons in the United States [5]. To identify BTD requests that pertained to an orphan drug, FDA’s Orphan Drug Designations and Approvals database [6] was reviewed to determine whether the indication and drug name from the request were included in the database as of May 12, 2020. Descriptions noted in the BTD review memo about the availability of therapy were assessed to determine if a therapy was available for the specific indication in the BTD request. An *available therapy* is generally interpreted as an FDA-approved/licensed therapy for the same indication being considered for the new drug and is relevant to current U.S. standard of care (SOC) for the indication. A drug would not be considered available therapy if the drug received accelerated approval based on a surrogate endpoint or an intermediate clinical endpoint and clinical benefit has not been verified by post-approval studies.

### Description of the Analysis

Analyses were performed and summarized descriptively as numbers and percentages, and general patterns are reported. Given the multiple factors involved with each BTD request, we do not attempt to establish causal relationships between individual request characteristics and a decision to grant or deny.

Information about the phase of the trial(s) supporting granted BTD requests was collected, mostly from the BTDDRT. If not documented in the review, the original request submitted by the sponsor was examined to identify the information about trial phase. The latest (i.e., highest) phase of trial development supporting the request was tabulated when apparent, but the phase was designated as *other* if information about phase was unavailable or not clearly delineated in the sponsor’s submission and in the CDER review.

Although various factors contributed to the decision to grant or deny each BTD request, for descriptive purposes the statistical significance of drug-outcome associations were one focus of the current analysis. Specifically, *p* values for the primary endpoint (or co-primary endpoints) for trials that were submitted in documents with the BTD request were reviewed. For some requests, more than one p value was recorded (e.g., for multiple dosages, multiple time points, various genetic subtypes) if the associations contributed to the BTD decision. Similarly, if a secondary endpoint was identified as especially relevant by the review division, the *p* value of the secondary endpoint was also collected.

While recognizing that each BTD request has unique attributes, granting of a BTD request indicates that the review team viewed the submitted evidence as satisfying relevant regulatory criteria. Conversely, and to characterize reasons for denial of BTD requests, this study examined information included in the determination letter issued to the sponsors. The corresponding reasons for denial were assigned to six categories (similar to the criteria described in the earlier CDER study [3]) and related to (1) insufficient (e.g., uncertain or too-small) treatment effect, (2) inadequate trial design, (3) too-small sample size or issues with data quality, (4) endpoint attributes, (5) safety issues, and (6) reliance on post hoc analysis. These factors are not mutually exclusive; interdependence exists among a number of these attributes, and some BTD requests were denied for more than one reason.

For the subset of BTD requests that were granted and subsequently rescinded or withdrawn, additional information was collected from CDER’s outgoing Intent to Rescind and Rescind letters, as well as incoming sponsors’ Withdrawal of BTD Requests.

Drug development programs for specific drugs and indications granted BTD between CY 2017 and CY 2019 that had been subsequently approved as of December 31, 2022, were identified. The number of days between granting of BTD and receipt of NDA/BLA was calculated as the difference between the date the BTD was granted and the NDA/BLA receipt date obtained from the approval letters.

## Results

### Therapeutic Characteristics of BTD Requests

Among 240 non-oncology BTD requests, 93 requests (39%) were granted and 147 requests (61%) were denied. Table 1 shows a summary of BTD decisions in therapeutic areas, including pulmonary, allergy, and rheumatology (14%); dermatology and dental (13%); psychiatry (13%); neurology (12%); gastroenterology and inborn errors (10%); and cardiology and renal (9%). Of note, four of these therapeutic areas were among the top five non-oncology therapeutic areas identified in a previous analysis conducted by CDER [3]. This analysis did not consider the number of BTDs granted relative to the number of active INDs in each area; accordingly, any differences observed may be related to the number of active development programs.

**Table 1:** BTD Decisions in 2017–2019 and Therapeutic Area

Product Type	Total (%)	Granted	Denied
Pulmonary, allergy and rheumatology	34 (14%)	13	21
Dermatology and dental	32 (13%)	8	24
Psychiatry	30 (13%)	11	19
Neurology	29 (12%)	6	23
Gastroenterology and inborn errors	24 (10%)	8	16
Cardiology and renal	22 (9.1%)	7	15
Hematology	16 (6.7%)	15	1
Antiviral	14 (5.9%)	11	3
Anesthesia, analgesia, and addiction	13 (5.4%)	3	10
Anti-infective	8 (3.3%)	5	3
Transplant and ophthalmology	8 (3.3%)	1	7
Metabolism and endocrinology	6 (2.5%)	4	2
Bone, reproductive, and urology	3 (1.3%)	1	2
Medical imaging	1 (0.4%)	0	1
Total	240	93 (39%)	147 (61%)

Additional analyses of the BTD requests examined whether an FDA-approved therapy was available for the proposed indication in the BTD request at the time of submission; see Table 2. Among 107 (45%) of the 240 BTD requests for which FDA-approved therapy was available at the time of BTD submission, 30 (28%) were granted and 77 (72%) were denied. Among the 133 (55%) of the 240 requests that were submitted for indications for which no approved therapies were available, 63 (47%) were granted and 70 (53%) were denied.

**Table 2:** BTD Decisions and Availability of FDA-Approved Therapy

Category; *n* (%)	Granted	Denied
Therapy available; *n* = 107 (45%)	30 (28%)	77 (72%)
No available therapy; *n* = 133 (55%)	63 (47%)	70 (53%)
Total; *n* = 240	93	147

The orphan-designation status of the BTD requested drugs is summarized in Table 3. Of note, this assessment did not discern whether the request pertained to an *orphan subset* of persons among those with the disease or condition involved. Among 240 BTD requests, 90 (42%) were for orphan drugs, of which 47 (52%) were granted and 43 (48%) were denied. In contrast, among 150 (58%) requests that were not for an orphan drug, 46 (31%) were granted and 104 (69%) were denied.

**Table 3:** BTD Decisions and Orphan Designation of the BTD Drugs

Category; *n* (%)	Granted	Denied
Orphan; *n* = 90 (42%)	47 (52%)	43 (48%)
Not orphan; *n* = 150 (58%)	46 (31%)	104 (69%)
Total; *n* = 240	93	147

Table 4 shows the combined pattern of orphan status of candidate drugs and availability of therapy for the respective indications for granted and denied BTD requests. The results show that 26% (*n* = 63) of the BTD requests were for drugs designated as orphan and with no available therapy; the rate of granting BTD requests is highest (56%) for this subset of requests. In contrast, among the 33% (*n* = 80) of BTD requests for drugs that are not designated as orphan and where therapy is available, the rate of granting the request was lowest (23%).

**Table 4:** BTD Decisions and Availability of FDA-Approved Therapy Combined with Orphan-Designation Status

Category; *n* (% of Total)	Granted	Denied
Orphan, no available therapy; *n* = 63 (26%)	35 (56%)	28 (44%)
Orphan, available therapy; *n* = 27 (11%)	12 (44%)	15 (56%)
Not orphan, no available therapy; *n* = 70 (29%)	28 (40%)	42 (60%)
Not orphan, available therapy; *n* = 80 (33%)	18 (22.5%)	62 (77.5%)
Total; *n* = 240	93	147

### Attributes of Granted BTD Requests

The 93 granted BTD requests were further evaluated to obtain information on (1) clinical characteristics of the supporting trials; (2) preliminary clinical evidence of substantial improvement over available therapy; (3) rationale for withdrawal of BTD requests or rescission of BTDs granted where applicable; and (4) marketing approval status.

Table 5 shows that the latest trial phase supporting a majority of the BTD requests was Phase 2 (*n* = 50) or Phase 3 (*n* = 15). Data from patients receiving candidate drugs under expanded access (EA) were included in four requests. In two of these requests, the EA data were used to supplement the data collected from clinical trials. In one request, for a very rare disease, EA data from one patient and limited information from another patient were used to support granting the request. In another case, also for a very rare disease, EA data from 13 patients were included to support the request for breakthrough therapy.

**Table 5:** Latest Phase of Trial Supporting Granted BTD Requests

Trial Phase*	# of Trials
Expanded access	4*
Phase 1	6
Phase 1/2	6
Phase 2	50
Phase 2/3	5
Phase 3	15
Phase 4	1
Other (not reported)	8

*In two BTD requests, data from expanded access was used to support other clinical trial data

The studies supporting granted BTD requests included at least one randomized trial in 71 (76%) of requests, the majority of which (*n* = 58; 82%) were blinded. Although data from blinded trials supporting BTD were primarily from double-blind studies (*n* = 53), single-blind (*n* = 4), and blinded-to-dose-strength (*n* = 1) trials were also noted. In 71 (76%) BTD requests, a placebo, vehicle, active control, or no treatment was used in a concurrent control group. In 22 (24%) of granted BTD requests without a control arm, study characteristics included use of historical or baseline controls (e.g., extrapolation from trials of approved indications or contemporaneous external controls).

As mentioned previously, among 63 (68%) of 93 granted BTD requests, no therapy was available for the indication for which the BTD was requested. For these requests, CDER’s decision to grant BTD was based most often on requests (*n* = 62) with specific data showing a clinically meaningful treatment effect on important outcomes, including six instances in which the effect size was reported as relatively small but was still considered meaningful because of, in part, the absence of available therapy. For one request related to medical countermeasures, the decision to grant BTD was based on extrapolation of data from a clinical trial of a related approved indication and data from animal studies, given that conducting a human clinical trial for the BTD requested indication was determined not to be appropriate due to ethical considerations.

For 30 (32%) of 93 granted BTD requests, therapy was already available for the indication for which BTD was requested. For these requests, CDER’s decision to grant BTD included consideration of (1) preliminary evidence that the BTD drug had the potential to be a substantial improvement over available therapy for the specified indication (*n* = 20); (2) efficacy of the BTD drug being comparable to available therapy that was known to be discontinued by the manufacturer (*n* = 2); (3) BTD drugs having an improved safety profile (*n* = 4); and (4) other (*n* = 4), including the BTD drug being intended for patients who do not respond adequately to the available therapy.

BTD requests were received for a wide variety of indications (e.g., cytomegalovirus infection, neurotrophic keratitis, sickle cell disease, weight management), and diverse clinical endpoints were used to provide preliminary clinical evidence of efficacy. Accordingly, the type of point estimate used to evaluate efficacy varied and included hazard ratios, relative or mean changes compared to control, change in least-square mean values, etc. This variability in diseases and clinical endpoints limited our ability to directly compare the preliminary clinical evidence and decisions made to grant or deny BTD across various therapeutic areas. Nonetheless, in 60 (65%) of 93 granted BTD requests, information about the statistical significance of the data supporting the requests was included in the BTD request review, and in 90%, the *p* value was less than 0.05 (including 57% < 0.01). In the remaining 10% of granted BTD requests, nominal statistical significance was noted for a key secondary endpoint (and the *p* value for the primary endpoint was typically less than 0.10).

BTD requests for which a p value was not reported or assessed (*n* = 33) most often involved orphan diseases. Among these requests, various attributes were observed, including a small number of patients receiving the drug (< 30, and often < 10), with such requests often having a summary-level external control.

Several other factors are noted in our review of the requests which may have influenced decision making by review divisions. For example, for nine granted BTD requests, the reviewers noted that the mechanism of action was either novel or complementary to existing treatment, and therefore, the potential for additional efficacy using combination therapy was recognized. In addition, preliminary evidence suggesting a sustained response to the drug was noted as supportive information for nine requests. Preliminary clinical evidence suggesting not only efficacy but also the potential for improved safety compared to available therapy, including less abuse potential, was mentioned for nine requests. In three cases, an apparent improvement in the safety profile was emphasized as a reason for granting the BTD.

Eight granted BTDs were subsequently rescinded by FDA for reasons including (1) approval of new therapies for the respective indications such that the BTD therapy was no longer found likely to show a substantial improvement over available therapy; (2) sponsor’s plan to discontinue the drug development program; and (3) additional data from clinical trials subsequent to BTD indicate that the drug is unlikely to demonstrate substantial improvement over available therapies.

When a BTD request is denied, the sponsor has the option to submit a new request to provide additional data. Among the 93 BTD requests that were granted, 8 (9%) were new submissions that were previously denied by FDA but were resubmitted with a revised indication or with additional data, to support the request.

Three BTD requests were withdrawn by the sponsor after being granted BTD. For one such request, the review division raised concerns about subsequent safety data that became available after BTD was granted, and the sponsor requested the withdrawal. For two other requests, the sponsor requested withdrawal of BTD per the advice of the review divisions. In one case, a collaborative decision was made to contemporaneously grant a new BTD for a broader indication for the same drug development program that encompassed the original BTD; in another case, the sponsor was asked to submit a new request under a new IND for the specified drug development program, and subsequently the original request was withdrawn. Overall, and excluding the granted BTD request that was withdrawn due to granting BTD for a broader indication, ten (11%) of 93 drugs did not retain the BTD designation.

As of December 31, 2022, 28 drugs involving 29 BTD requests that were granted BTD between calendar years 2017 and 2019 received marketing approval for the indication for which BTD was granted; see Table 1, Supplementary Materials. Of note, for 11 drugs the NDA/BLA was received within a year after the corresponding BTDs were granted, indicating that some requests were granted relatively late in drug development. Twenty one of these 28 drugs (75%) are *first in class*, which indicates that the drugs have mechanisms of action different from those of existing therapies.

### Attributes of Denied BTD Requests

The BTD requests denied without review by the MPPRC (*n* = 61; 42%) were denied due to clearly not aligning with BTD criteria, as discussed in the “Methods” section. Examples include instances where the indication was not a serious or life-threatening disease, the participants enrolled in the trial were not representative of the patient population for which BTD was being sought, or demonstration of potential improvement over available therapy was lacking. The current analysis focused on the 86 BTD requests denied by the review division after taking specific feedback from the MPPRC into consideration. In 26 (30%) of 86 denied requests, a single reason was cited for denial. In the remaining 60 (70%) denied requests, the decision to deny was based on multiple reasons. Reasons for denial are shown in Table 6, with insufficient treatment effect identified as the most frequent reason for denial (*n* = 50; 58%). The specific examples included small, or no, treatment effect observed compared to an appropriate comparator; lack of statistical significance for the effectiveness endpoint, when applicable; pattern of dose response that raised uncertainty about efficacy; and loss of efficacy over time.

**Table 6:** Reasons for Denial (*n* = 86) of BTD Requests (Not Mutually Exclusive)

Reason for Denial	*n* (%)
Insufficient treatment effect	50 (58%)
Inadequate trial design	46 (53%)
Data-related issues	33 (38%)
Endpoint attributes	28 (33%)
Safety issues	14 (16%)
Reliance on post hoc analysis	5 (6%)

As shown in Table 6, aspects of trial design were the second most common (*n* = 46; 53%) reason for denial, including problems related to short study duration making it difficult to assess preliminary evidence of substantial benefit, lack of an appropriate control arm, concern regarding multiplicity when secondary endpoints were being relied on, and an external control group matched poorly to patients in a single-arm trial. In 33 (38%) of denied BTD requests, data-related issues were cited as reasons for denial. Corresponding examples, whether as a single reason or multiple reasons, included lack of trial participants’ pre-treatment history, timing of improvement incongruent with anticipated mechanism of drug, or inadequate data regarding the comparator arm.

As another reason (*n* = 28; 33%) for denial, the review division determined that the endpoint was not clinically relevant for patients with the disease. Examples of endpoint issues identified include a lack of supportive evidence that the endpoint used by the sponsor predicted a clinically meaningful benefit, or the endpoint did not represent a serious aspect of the disease.

Safety was cited as a reason for denial in 14 (16%) requests. An example in this category includes an excess of adverse events in the investigational arm versus the control arm. Flawed (e.g., unblinded) post hoc analyses were noted as a reason for denial in five requests (6%). An in-depth description of the various characteristics (e.g., phase, blinding) of the trials for denied requests was not conducted.

## Discussion

A previous analysis was conducted by CDER on 364 BTD requests in all therapeutic areas received from the BTD program inception in 2012 through June of 2016 [3]. The current analysis focuses on an evaluation of non-oncology BTD requests for which the decision to grant or deny the request was made in CY 2017–2019. Oncology studies often involve similar endpoints across different types of cancer, representing an attribute that can aid in consistency, whereas this study analyzed diverse non-oncology therapeutic areas where evaluating consistency is more challenging.

The current evaluation differs from the previous evaluation [3] due to exclusion of oncologic drugs, restructuring of review divisions after the previous evaluation, and inherent variability in BTD requests submitted in any given time period. In addition, detailed data regarding all denied BTD requests were included in the previous analysis, whereas detailed data regarding BTD requests denied only after receiving MPPRC feedback are included in the current analysis. For these reasons, direct comparisons of results between the two reports are unwarranted.

Although most of the data for these analyses were collected directly from CDER’s and sponsors’ documents, categorization of information (e.g., reasons for denying the requests) was needed to present results in a cohesive format. Such categorization can at times be subject to interobserver variability, but the primary and secondary reviewers discussed challenging scenarios to reach consensus. When evaluating these BTD requests received by CDER across diverse non-oncology therapeutic areas, various patterns were evident. As expected, based on the criteria for BTD, granted requests were for serious or life-threatening conditions with the supportive data deemed to be reliable as well as relevant and study designs considered adequate, whereas inadequate preliminary evidence of substantial improvement over existing therapies is the statutory criteria for which requests are denied. In addition, as shown in Table 4, BTD is more often granted for requests involving orphan diseases where no therapy is available, suggesting that the BTD program is supporting development of orphan drugs for which there is unmet need. This pattern may also reflect a general increase in applications for orphan diseases [7].

Most data supporting granted BTD requests were derived from Phase 2 or Phase 3 trials. In general, the trials were randomized and blinded. As reflected by the focus in reviews of BTD requests on attributes of trial design and the strength of findings involved—and despite the clinical contexts of such requests varying widely—CDER used a structured approach when assessing whether the study was adequate to demonstrate substantial improvement over available therapy. Of note, statistical significance (or lack thereof) was not a necessary criterion for granting (or denying) BTD. As noted previously, one of the criteria for granting BTD is that preliminary clinical evidence indicates that the drug may demonstrate substantial improvement over existing therapies. More detailed analyses (e.g., specific statistical approaches or what data sources were used to serve as external control arms for single-arm trials) were beyond the scope of this work.

Not all BTD drug development programs were able to retain *granted* status as development progressed. Reasons for rescission and withdrawal after granting indicate that a subset of drugs failed to deliver the potential benefits anticipated, a new drug was approved for the same indication which made the BTD drug development program no longer better than available therapy, or a sponsor determined not to pursue their BTD drug development program, all of which highlight the inherent uncertainties of clinical developmental programs at the time a BTD is granted.

A BTD provides several benefits to the sponsor, including increased frequency of meetings with the FDA, enhanced communications during drug development, an organizational commitment to involve senior-level managers in the development of the drug for a specific indication, assignment of a cross-disciplinary project lead, and eligibility for rolling review. This paper focuses on how BTD requests are reviewed and either granted or denied, and not the benefits that a sponsor is afforded once a BTD is granted. Nonetheless, and regarding the process for making a determination on BTD requests, this analysis indicates that CDER’s BTD program is implemented in a manner consistent with Section 506(a) of the Federal Food, Drug, and Cosmetic Act (as added by Section 902 of FDASIA) and the principles outlined in FDA guidance on expedited programs [1] to enhance and potentially expedite development of promising therapies for serious conditions.

### Supplementary Information

Below is the link to the electronic supplementary material.

Supplementary file1 (PDF 181 kb)

## Data Availability

Not applicable.

## References

[1] U.S. Food and Drug Administration. Guidance for industry. Expedited programs for serious conditions-drugs and biologics. 2014.

[2] U.S. Congress. Food and Drug Administration Safety and Innovation Act (FDASIA). 2012.

[3] Conrad R, Taylor K, Raggio M (2017). Breakthrough therapy designation: CDER analysis of requests 4 years into the program. Ther Innov Regul Sci.

[4] IND regulations, 21 CFR §312.130. 1987.

[5] Orphan drug regulations, 21 CFR §316. 2011.

[6] U.S. Food and Drug Administration. Orphan drug designations and approvals database Available from: https://www.accessdata.fda.gov/scripts/opdlisting/oopd/index.cfm. Accessed 31 Oct 2023.

[7] CDER. Advancing health through innovation: new drug therapy approvals 2022. Available from: https://www.fda.gov/drugs/new-drugs-fda-cders-new-molecular-entities-and-new-therapeutic-biological-products/novel-drug-approvals-2022. Accessed 31 Oct 2023.

