# Supplementary material for: Decisions on Non-oncology Breakthrough Therapy Designation Requests in 2017–2019
Source: Ther Innov Regul Sci. 2023 Nov 5;58(1):214–21. doi: 10.1007/s43441-023-00589-z (PMC10764372; doi:10.1007/s43441-023-00589-z)
Supplement: Supplementary file 1 — Supplementary file1 (PDF 181 kb) [file 43441_2023_589_MOESM1_ESM.pdf]

Supplementary Table 1: Time from BTD to receipt of marketing application through calendar year (CY) 2022 for BTDs granted in CY 2017-2019.

Proprietary Name (Established Name)	Days Between Granting BTD & Receipt of NDA/BLA	NDA/BLA Indication Approved	First in Class?
PREVYMIS (Letermovir)	19	Prophylaxis of cytomegalovirus (CMV) infection and disease in adult CMV-seropositive recipients [R+] of an allogeneic hematopoietic stem cell transplant (HSCT)	Yes
OXERVATE (cenegermin-bkbj)	22	Treatment of neurotrophic keratitis	Yes
ONPATTRO (Patisiran)	24	Treatment of the polyneuropathy of hereditary transthyretin-mediated amyloidosis in adults	Yes
UPLIZNA (inebilizumab-cdon)	55	Indicated for the treatment of neuromyelitis optica spectrum disorder (NMOSD) in adult patients who are anti-aquaporin-4 (AQP4) antibody positive	Yes
ADAKVEO (Crizanlizumab-tmca)	147	To reduce the frequency of vasoocclusive crises in adults and pediatric patients aged 16 years and older with sickle cell disease	Yes

Proprietary Name (Established Name)	Days Between Granting BTD & Receipt of NDA/BLA	NDA/BLA Indication Approved	First in Class?
VYNDAQEL (Tafamidis meglumine)	168	Treatment of the cardiomyopathy of wild type or hereditary transthyretin-mediated amyloidosis in adults to reduce cardiovascular mortality and cardiovascular-related hospitalization	Yes
INMAZEB (atoltivimab, maftivimab, and odesivimabebgn)	175	Indicated for the treatment of infection caused by Zaire ebolavirus in adult and pediatric patients, including neonates born to a mother who is RT-PCR positive for Zaire ebolavirus infection	Yes
ENSPRYNG (Satralizumab-mwge)	240	Treatment of neuromyelitis optica spectrum disorder (NMOSD) in adult patients who are anti-aquaporin-4 (AQP4) antibody positive	Yes
TZIELD (Teplizumab-mzwv)	256	To delay the onset of Stage 3 type 1 diabetes (T1D) in adults and pediatric patients aged 8 years and older with Stage 2 T1D	Yes
EBANGA (ansuvimab-zykl)	266	Treatment of infection caused by Zaire ebolavirus in adult and pediatric patients, including neonates born to a mother who is RT-PCR positive for Zaire ebolavirus infection	No

Proprietary Name (Established Name)	Days Between Granting BTD & Receipt of NDA/BLA	NDA/BLA Indication Approved	First in Class?
COSELA (trilaciclib)	319	Decrease the incidence of chemotherapy-induced myelosuppression in adult patients when administered prior to a platinum/etoposide-containing regimen or topotecan-containing regimen for extensive-stage small cell lung cancer	Yes
(Artesunate)	380	Initial treatment of severe malaria in adult and pediatric patients	No
TRIKAFTA (Elexacaftor, tezacaftor, and ivacaftor; ivacaftor (co-packaged))	430	Treatment of cystic fibrosis (CF) in patients aged 12 years and older who have at least one F508del mutation in the CFTR gene	No
ZOKINVY (Lonafarnib)	464	To reduce the risk of mortality in Hutchinson-Gilford Progeria Syndrome (HGPS) in patients 12 months of age and older with body surface area of $0.39m^2$ and above, for the treatment of processing-deficient Progeroid Laminopathies with either heterozygous LMNA	Yes

Proprietary Name (Established Name)	Days Between Granting BTD & Receipt of NDA/BLA	NDA/BLA Indication Approved	First in Class?
		mutation with progerin-like protein accumulation or homozygous or compound heterozygous ZMPSTE24 mutations	
LIVMARLI (maralixibat)	464	For the treatment of cholestatic pruritus in patients with Alagille syndrome (ALGS) 1 year of age and older	No
OXBRYTA (Voxelotor)	539	Treatment of sickle cell disease in adults and pediatric patients 12 years of age and older	Yes
AUVELITY (Dextromethorphan hydrobromide and Bupropion hydrochloride)	700	Treatment of major depressive disorder (MDD) in adults	No
GIVLAARI (givosiran)	742	Treatment of adults with acute hepatic porphyria	Yes

Proprietary Name (Established Name)	Days Between Granting BTD & Receipt of NDA/BLA	NDA/BLA Indication Approved	First in Class?
SUNLENCA[1] (Lenacapavir)	760	Treatment of human immunodeficiency virus type 1 (HIV-1) infection in heavily treatment-experienced adults in combination with other antiretroviral(s)	Yes
OXLUMO (Lumasiran)	770	Treatment of primary hyperoxaluria type 1 (PH1) to lower urinary oxalate levels in pediatric and adult patients	Yes
CIBINQO (abrocitinib)	930	For the treatment of adults with refractory, moderate-to-severe atopic dermatitis whose disease is not adequately controlled with other systemic drug products, including biologics, or when use of those therapies is inadvisable	No
ENJAYMO (sutimlimab-jome)	1031	Decrease the need for red blood cell (RBC) transfusion due to hemolysis in adults with cold agglutinin disease	Yes
IMCIVREE (Setmelanotide)	1061	Chronic weight management in adult and pediatric patients 6 years of age and older with obesity due to leptin receptor (LEPR) deficiency	Yes

[1] Two new drugs applications for the oral and injectable formulations

Proprietary Name (Established Name)	Days Between Granting BTD & Receipt of NDA/BLA	NDA/BLA Indication Approved	First in Class?
		confirmed by genetic testing demonstrating variants in LEPR genes that are interpreted as pathogenic, likely pathogenic, or of uncertain significance (VUS)	
IMCIVREE Setmelanotide	1061	Chronic weight management in adult and pediatric patients 6 years of age and older with obesity due to proopiomelanocortin (POMC) or proprotein convertase subtilisin/kexin type 1 (PCSK1) deficiency or leptin receptor (LEPR) deficiency confirmed by genetic testing demonstrating variants in POMC, PCSK1, or LEPR genes that are interpreted as pathogenic, likely pathogenic, or of uncertain significance (VUS)	Yes
EVKEEZA (Evinacumab-dgnb)	1185	An adjunct to other low-density lipoprotein-cholesterol (LDL-C) lowering therapies for the treatment of adult and pediatric patients, aged 12 years and older, with homozygous familial hypercholesterolemia (HoFH)	Yes

Proprietary Name (Established Name)	Days Between Granting BTD & Receipt of NDA/BLA	NDA/BLA Indication Approved	First in Class?
LIVTENCITY (Maribavir)	1194	Treatment of adults and pediatric patients 12 years of age and older and weighing at least 35 kg with post-transplant cytomegalovirus (CMV) infection/disease that is refractory to treatment (with or without genotypic resistance) with ganciclovir, valganciclovir, cidofovir, or foscarnet.	Yes
KORSUVA (Difelikefalin)	1281	Treatment of moderate-to-severe pruritus associated with chronic kidney disease (CKD-aP) in adults undergoing hemodialysis (HD)	Yes
INCIVREE (Stemelanotide)	1599	For chronic weight management in adult and pediatric patients 6 years of age and older with Bardet-Biedl Syndrome (BBS)	No

.
